# Assessing the effects of intratendinous genipin injections: Mechanical augmentation and spatial distribution in an *ex vivo* degenerative tendon model

**DOI:** 10.1371/journal.pone.0231619

**Published:** 2020-04-15

**Authors:** Timo Tondelli, Tobias Götschi, Roland S. Camenzind, Jess G. Snedeker

**Affiliations:** 1 Department of Orthopedics, Balgrist University Hospital, Zurich, Switzerland; 2 Institute for Biomechanics, ETH Zurich, Zurich, Switzerland; University of Rochester, UNITED STATES

## Abstract

**Background:**

Tendinopathy is a common musculoskeletal disorder and current treatment options show limited success. Genipin is an effective collagen crosslinker with low cytotoxicity and a promising therapeutic strategy for stabilizing an intratendinous lesion.

**Purpose:**

This study examined the mechanical effect and delivery of intratendinous genipin injection in healthy and degenerated tendons.

**Study design:**

Controlled laboratory study

**Methods:**

Bovine superficial digital flexor tendons were randomized into four groups: Healthy control (N = 25), healthy genipin (N = 25), degenerated control (N = 45) and degenerated genipin (N = 45). Degeneration was induced by Collagenase D injection. After 24h, degenerated tendons were subsequently injected with either 0.2ml of 80mM genipin or buffer only. 24h post-treatment, samples were cyclically loaded for 500 cycles and then ramp loaded to failure. Fluorescence and absorption assays were performed to analyze genipin crosslink distribution and estimate tissue concentration after injection.

**Results:**

Compared to controls, genipin treatment increased ultimate force by 19% in degenerated tendons (median control 530 N vs. 633 N; p = 0.0078). No significant differences in mechanical properties were observed in healthy tendons, while degenerated tendons showed a significant difference in ultimate stress (+23%, p = 0.049), stiffness (+27%, p = 0.037), work to failure (+42%, p = 0.009), and relative stress relaxation (-11%, p < 0.001) after genipin injection. Fluorescence and absorption were significantly higher in genipin treated tendons compared to control groups. A higher degree of crosslinking (+45%, p < 0.001) and a more localized distribution were observed in the treated healthy compared to degenerated tendons, with higher genipin tissue concentrations in healthy (7.9 mM) than in degenerated tissue (2.3 mM).

**Conclusion:**

Using an *ex-vivo* tendinopathy model, intratendinous genipin injections recovered mechanical strength to the level of healthy tendons. Measured by genipin tissue distribution, injection is an effective method for local delivery.

**Clinical relevance:**

This study provides a proof of concept for the use of intratendinous genipin injection in the treatment of tendinopathy. The results demonstrate that a degenerated tendon can be mechanically augmented by a clinically viable method of local genipin delivery. This warrants further in vivo studies towards the development of a clinically applicable treatment based on genipin.

## 1 Introduction

According to estimates, one in four adults will be affected by a tendon disorder during their lifetime.[1–4] In addition to patient suffering from disability, dysfunction and pain, tendon injuries generate substantial costs to the healthcare system.[2,5,6] The accumulation of microtrauma by repetitive overloading exceeds the maximum healing capacity of the tendon and induces a pathological response leading to hypercellularity, collagen matrix disruption, an increased proteoglycan content and neovascularization.[7,8] Tendinopathic lesions often do not resolve by natural healing and intervention is required in many cases to prevent lesion progression and restore tissue function.[9–11] The rotator cuff, forearm extensors, biceps brachii and tibialis posterior, patella and Achilles tendons are most commonly affected.[3,5]

A wide range of treatment options exists such as rest, physical therapy, nonsteroidal anti-inflammatory drugs and injection of glucocorticoids or platelet rich plasma (PRP).[12–14] Even though some options offer good short-term improvements, long-term benefits are limited and results are inconclusive.[15–21] Strengthening exercises improve symptoms and muscle-tendon function, but may in turn increase the risk for further mechanical damage.[22] Whereas most of these therapies aim to improve the healing capacity of the tissue, an alternative approach is to augment the mechanical strength of the affected tendon in order to prevent further damage accumulation.[23] Due to its low cytotoxicity, genipin (GP), a naturally occurring collagen cross-linking agent, is regarded as a promising candidate for tendon mechanical augmentation. In vivo intratendinous administration of high GP concentration in horses showed no local or systemic toxicity.[24] Incubation of animal tendon explants in 20 mM GP-solution improved suture retention strength by 30% after 24 hours[25] and successfully mitigated propagation of partial tendon tears[26]. In an ex-vivo model for tendon rupture repair, GP-coated sutures showed an increased suture retention strength by localized tissue strengthening when compared to uncoated sutures [27]. To date, the potential of GP in the repair of degenerative tendon lesions is still unknown. A key obstacle to clinical implementation is that no clinically viable method of localized GP application has yet been demonstrated for collagen-cross-linking based treatment of this indication. Therefore, this ex vivo laboratory study was performed in order to investigate the viability of intra-tendinous GP-injection to improve mechanical resistance of degenerated tendon tissue. Applied to chemically degenerated tendons, we hypothesized that GP injection increases ultimate tensile strength by locally crosslinking and repairing the damaged tissue. By making use of the distinct optical spectral properties (fluorescence, absorption) of GP-induced collagen-crosslinking, we analyzed the spatial GP distribution after intratendinous injection. In a separate set of experiments, we determined the relationship between GP incubation-concentration and induced fluorescence.

## 2 Materials and methods

**Fig 1:**
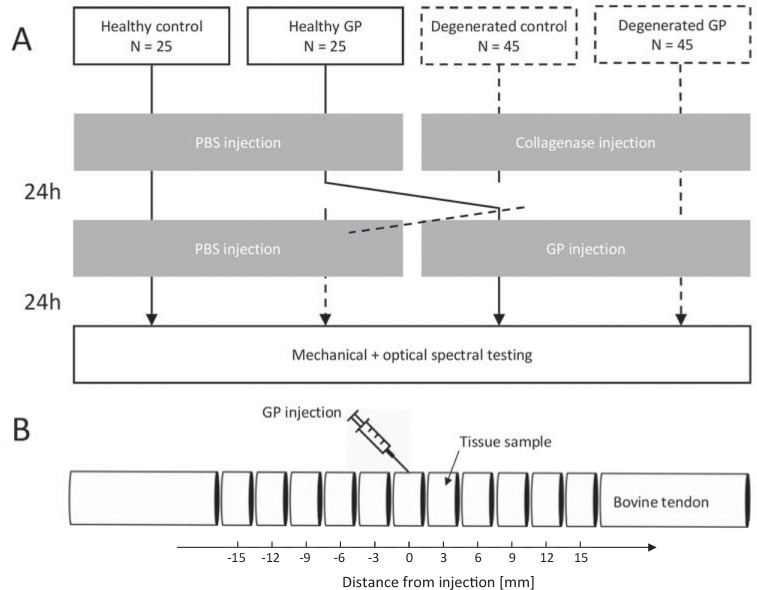
A: Study design and timeline of the main experiment. B: Cubes represent the sites of tissue sample harvesting for optical spectral testing.

A total of 70 bovine superficial digital flexor tendons (SDFT) were obtained from a local abattoir (Mean age 14 months; Abattoir: Metzgerei Angst AG, Zürich, Switzerland). Freshly harvested tendons were wrapped in gauze, moistened with phosphate buffered saline (PBS) solution and stored at -20°C until testing. At the day of testing the tendons were thawed and cut in half. Then the cross sectional area at the central part of the tendon was determined using a custom made laser based measuring device. Subsequently, each half was randomized into one of four groups: Healthy control (N = 25), healthy GP injection (N = 25), degenerated control (N = 45) and degenerated GP injection (N = 45). An overview of the experiment setup is provided in Fig 1A. The institutional review board approved the animal protocol (non-live tissue) for this investigation and that all investigations were conducted in conformity with ethical principles of research.

### 2.1 Tendon repair

On the first day of testing, the degenerated testing groups were injected centrally with 0.2 ml of collagenase D in PBS (8mg/ml, Collagenase D type Ref Number 11088866001, Roche Diagnostics GmbH, Rotkreuz, Switzerland) to disrupt the collagen matrix and model a degenerative tendon lesion.[28,29] The tendon degeneration protocol was established in a preliminary experiment. Both healthy groups were injected with 0.2ml PBS at the same point in time. Thereafter, all explants were wrapped in gauze, moistened with PBS and stored at room temperature for 24h until repair.

At 24 hours after the introduction of collagenase, the tendon specimens were injected centrally with either 0.2 ml PBS-DMSO (control groups, 2% DMSO) or 0.2 ml of 80 mM GP in a PBS-DMSO solution (treatment groups). The GP treatment solution contained 80mM GP (Challenge Bioproducts Co, Ltd, Taiwan, Republic of China) and 2% of the solvent dimethyl sulfoxide (DMSO, Sigma-Aldrich, St Louis, MO, USA) in PBS.

After the second treatment, all samples were wrapped in moist gauze (PBS) and stored at room temperature for 24h until mechanical testing. All injections were performed with a 24G needle.

### 2.2 Mechanical testing

Mechanical testing was performed on day three of the experiment. Both ends of the tendon were wrapped in pieces of cloth and glued with cyanoacrylate to prevent slippage. The samples were kept moist by PBS spraying for the duration of mechanical testing. The tendon ends were fixed to a 20 kN load cell (Gassmann Theiss, Bickenbach, Germany) of a universal material testing machine (Zwick 010, Zwick GmbH, Ulm, Germany) using dedicated clamps at a clamp-to-clamp distance of 45mm. After applying a preload of 0.1 MPa for one minute the specimens were cyclically elongated from 0% to 5% strain for 500 cycles with a frequency of approximately 1 Hz. Finally, the explants were loaded to failure at 15% strain/s. Force (N) and displacement (mm), time (s) were recorded at a sampling frequency of 100 Hz with a dedicated software (testExpert^®^ 10, Zwick-Roell, Ulm, Germany). Displacement was measured from clamp to clamp. The mode of failure was documented for each test. One specimen was removed due to a preexisting laceration incurred during sample preparation.

### 2.3 Optical spectral properties

Subsequent to mechanical testing, 36 tendons (9 from each group) were randomly selected for optical spectral testing. The required samples were determined by a priori power analysis (t-test). From each selected explant, 7 tissue biopsies measuring approximately 3 mm by 3 mm were harvested as highlighted in Fig 1B. The tendon extracts were lyophilized for 6h (Alpha 2–4 LSCplus, Martin Christ Gefriertrocknungsanlagen GmbH, Osterode am Harz, Germany) and tissue dry weight was recorded. Thereafter, extracts were digested in 0.6mg/mL papain (0.9 mL per sample; Sigma-Aldrich, St. Louis, MO, USA) in PBE buffer (100 mmol/L phosphate, 10 mmol/L EDTA, pH 6.5) at 65°C for 72 h.[30] Subsequently, the tissue extracts were centrifuged for one hour at 8000 relative centrifugal force (RCF). Each centrifuged sample was divided into three 0.2 mL aliquots on a 96 cell culture well-plate (Thermo Fisher Scientific, Waltham, MA, USA). These aliquots were diluted at a ratio of 1:7 with de-ionized water to reach optimal light transmittance. Fluorescence was recorded at 590 nm excitation and 645 nm emission wavelength (SpectraMax GeminiXS, Molecular Devices, LLC., Sunnyvale, CA, USA).[31–34] Absorption was measured with a spectrophotometer (Epoch, BioTek Instruments GmbH, Luzern, CH) for wavelengths from 380 to 700nm at 10 nm increments, with peak absorption at 590 nm. Fluorescence and absorption readouts were normalized by dry weight.

### 2.4 Experiment GP concentration-fluorescence intensity relationship

In a separate set of experiments, the relationship between GP induced fluorescence and GP concentration was established. A total of 75 tissue samples measuring approximately 3 mm by 3 mm were extracted from two bovine SDFTs. The samples were randomly assigned to be incubated for 24 hours in either a 0, 0.5, 1, 2, 5, 10, 20, or 40 mM GP-PBS solution (10 samples per group, 5 samples in the control group). The desired GP concentrations were obtained by diluting a solution containing 80mM GP (Challenge Bioproducts Co, Ltd, Taiwan, Republic of China) and 2% of the solvent dimethyl sulfoxide (DMSO, Sigma-Aldrich, St Louis, MO, USA) in PBS. Lyophilization, dry weight recording, digestion, centrifugation and fluorescence measurement was performed identically to the main experiment and are described above.

### 2.5 Data analysis

Ultimate force, ultimate stress and strain were defined at the maximum load reached. Stiffness and the elastic modulus was defined as the maximum gradient obtained from a series of linear regressions (*Force = a+b∙displacement*, ordinary least squares (OLS)) from the end of preload up to maximum load. Work to failure was computed from the end of preload up to maximum load. Relative stress relaxation was defined as the relative drop in maximum cyclic force from the first to the last tested cycle. An a priori power analysis (independent samples t-test) using the results of the degeneration protocol experiment and a minimally important difference of 20% in ultimate force yielded a required sample size of 23 and 43 for healthy and degenerated tendons, respectively.[25, 43] Normality assumption was rejected by the Shapiro-Wilk test for some mechanical variables. Hence, Kruskal-Wallis and Dunn’s multiple median comparison test were used for inter-group comparison of mechanical data.

Normality was not rejected for fluorescence and absorption. Difference from zero was tested by t-tests. To analyze the difference at the injection site across groups, a linear regression (estimated by ordinary least squares, OLS) with dependent variable being fluorescence and absorption and independent variables a dummy for each group was estimated. Another regression was used to compare spatial distribution of GP across the treatment groups, which is highlighted in Eq 1.

$$\begin{array}{l} Y=\beta_{0}+\beta_{1}∙D_{ColGP}\\ \;+\beta_{9mm}^{GP}∙D_{9mm}∙(1-D_{ColGP})+\beta_{15mm}^{GP}∙D_{15mm}∙(1-D_{ColGP})\\ \;+\beta_{9mm}^{ColGP}∙D_{9mm}∙D_{ColGp}+\beta_{15mm}^{ColGP}∙D_{15mm}∙D_{ColGp} \end{array}$$ (1)

The dependent variables were fluorescence and absorption (Y). A separate regression was estimated for each dependent variable. The independent variables were a dummy for healthy (1−*D_ColGP_*) and degenerated GP (*D_ColGp_*), and dummy interaction terms for each group and distance from injection site (*D*_9*mm*_,*D*_15*mm*_). The injection site was used as baseline, the interaction coefficients ($\beta_{9mm}^{GP};\;\beta_{15mm}^{GP};\;\beta_{9mm}^{ColGP};\;\beta_{15mm}^{ColGP}$) show the decrease in fluorescence or absorption for each group at a certain distance.

Finally, the baseline relationship between GP induced fluorescence and GP concentration was assessed by estimating a linear regression model (OLS) with fluorescence (excitation 590 nm, emission 645 nm) as dependent and the natural logarithm of GP concentration as independent variable. The obtained equation was subsequently used with input factors being fluorescence and absorption at the injection site to estimate GP concentration in the main experiment. The significance level was set at 0.05 and the results are reported as medians and range if not stated otherwise. The statistical analyses and graphs were computed using MATLAB (MATLAB and Statistics Toolbox Release 2016b, The MathWorks, Inc., Natick, Massachusetts, USA) and Stata 14.0 (StataCorp LP, College Station, TX, USA).

## 3 Results

### 3.1 Mechanical effects of genipin injection

**Fig 2:**
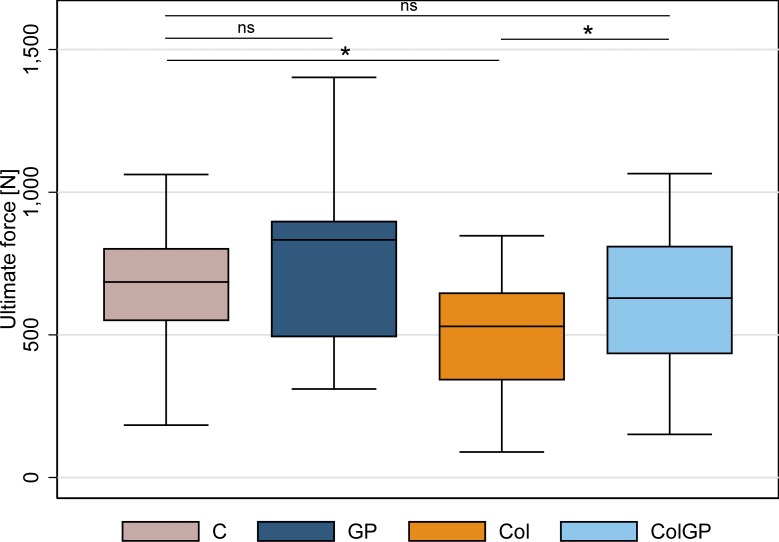
Box plots of ultimate force [N] across groups. Group notation: C healthy control; GP healthy GP-treated; Col degenerated control; ColGP degenerated GP-treated. Median comparison by Dunn’s test. * denotes p-value≤0.05, ns denotes not significant with p>0.05.

All tendon samples survived cyclic testing and ruptured at the midportion during ramp-to-failure testing with no failure due to slippage being observed. Table 1 summarizes the effects of GP treatment on the mechanical properties of healthy and degenerated tendons. Fig 2 depicts the ultimate force to failure across groups. Cross sectional area (CSA) was homogenously distributed among all groups (p = 0.137). GP treatment augmented maximum tendon rupture force (ultimate force) in healthy (+22%, p = 0.325) and in degenerated tendons (+19%, p = 0.008). In healthy tendons median ultimate force after 24 h of GP and PBS (healthy control) treatment was 833 N and 685 N, respectively.

**Table 1:** Results of mechanical testing for each group with the respective differences. P-values are two-sided and based on a nonparametric pairwise comparison of multiple groups (median, Dunn’s test). Stiffness and elastic modulus were calculated as the maximum gradient obtained from a series of linear regressions on the force-displacement curve.

	**Healthy**					
	Control (N = 25)		GP-Treated (N = 25)			
	Median	(Range)	Median	(Range)	Difference	p-value
Ultimate force [N]	685	(184–1062)	833	(310–1403)	22%	0.325
Ultimate stress [MPa]	25.6	(6.4–41.8)	34.7	(9.7–66.8)	36%	0.272
Stiffness [N/mm]	152	(29–282)	218	(44–314)	43%	0.245
Elastic modulus [MPa]	193	(53–497)	237	(48–622)	23%	0.469
Work to failure [mJ]	2304	(842–4133)	2577	(1151–4671)	12%	0.567
Strain at failure []	0.22	(0.13–0.59)	0.27	(0.13–0.55)	23%	0.219
Relative stress relaxation []	-0.66	(-0.88 - -0.48)	-0.49	(-0.72 - -0.39)	-26%	0.025
CSA [mm^2^]	26.4	(16.8–35.1)	25.3	(17–39.8)	-4%	0.493
	**Degenerated**					
	Control (N = 45)		GP-Treated (N = 44)			
	Median	(Range)	Median	(Range)	Difference	p-value
Ultimate force [N]	530	(89–848)	633	(151–1065)	19%	0.008
Ultimate stress [MPa]	18.4	(4.4–52.7)	22.7	(6.7–38.8)	23%	0.049
Stiffness [N/mm]	111	(17–222)	141	(25–290)	27%	0.037
Elastic modulus [MPa]	137	(27–526)	170	(53–321)	24%	0.285
Work to failure [mJ]	1612	(172–3278)	2294	(446–4847)	42%	0.009
Strain at failure []	0.28	(0.12–0.6)	0.29	(0.12–0.6)	4%	0.701
Relative stress relaxation []	-0.88	(-0.98 - -0.05)	-0.78	(-0.94 - -0.45)	-11%	0.000
CSA [mm^2^]	27.8	(11.9–43.2)	28.5	(17.8–42.2)	3%	0.537

This difference of +148 N was statistically non-significant (p = 0.325). In the degenerated tendons, GP treatment improved ultimate force from 530 N in the untreated tendons to 633 N. In this case, GP showed a statistically significant therapeutic effect of +103 N (p = 0.008) on degenerated tendons. While untreated degenerated tendons ruptured at a lower force compared to the healthy controls (-155 N, p = 0.004), no statistically significant difference was detectable after GP treatment of degenerated tendons and the healthy controls (-52N, p = 0.521). Compared to ultimate force, GP treatment had a similar effect on other mechanical properties of the tendons. In healthy tendons GP injected samples had higher ultimate stress (+36%, p = 0.272), stiffness (+43%, p = 0.245), elastic modulus (+23%, p = 0.469), and work to failure (+12%, p = 0.567) compared to the controls, however, these differences were statistically not significant. Relative stress relaxation was the only value that was reduced by GP injection in healthy tendons (—26%, p = 0.025). In contrast to healthy tendons, the degenerated tendons showed a statistically significant GP induced improvement in all measured biomechanical variables. Ultimate stress was improved by +23% (p = 0.049), stiffness by +27% (p = 0.037), work to failure by +42% (p = 0.009) and relative stress relaxation by -11% (p < 0.001). The increase in elastic modulus +24% (p = 0.285) and strain at failure +4% (p = 0.701) were statistically non-significant in degenerated tendons. Furthermore, GP injection recovered degenerated tendons to an extent that there was no statistically significant difference detectable compared to healthy controls for ultimate force (-8%, p = 0.521) ultimate stress (-11%, p = 0.176), stiffness (-7%, p = 0.359), elastic modulus (-12%, p = 0.083), and work to failure (0%, p = 0.541), and strain at failure (+32%, p = 0.157). Relative stress relaxation was +18% higher in GP treated degenerated tendons (p = 0.008) than in the healthy control group. When discussing statistically non-significant differences between groups it is important to keep in mind the properties of a-priori sample size calculation. In particular for non-significant differences between the healthy groups, which were as high as 43% (stiffness), and could be due to insufficient power retrospectively.

### 3.2 Optical spectral properties

**Fig 3:**
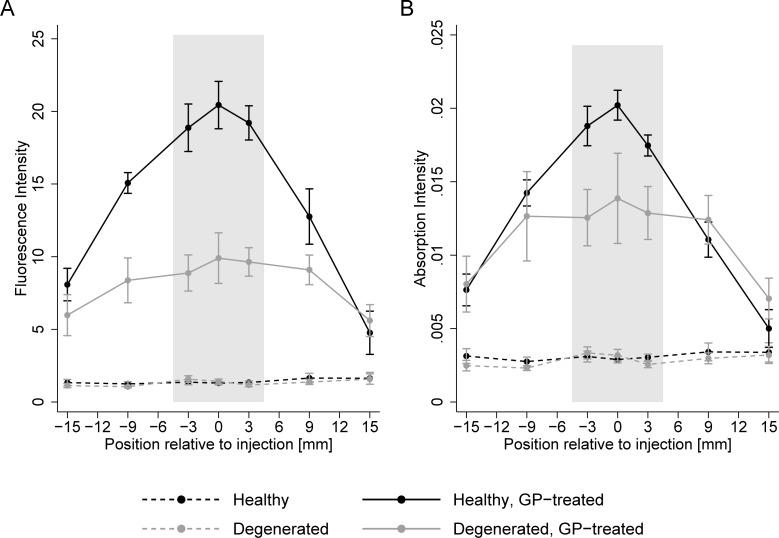
A: Mean fluorescence intensity at excitation wavelength 590 nm and emission wavelength 645 nm for each group. Bars denote standard errors. Values are dry weight normalized. The gray area depicts the tendons’ central part (+/- 4.5 mm from the injection site) B: Mean absorption intensity at wavelength 590 nm for each group. Bars denote standard errors. Values are dry weight normalized. The gray area depicts the tendons’ central part (+/- 4.5 mm from the injection site).

As highlighted in Fig 3, GP specific fluorescence (excitation and emission wavelength are 590nm and 645nm, respectively) at the injection site was higher in healthy GP treated tendons compared to healthy control, degenerated control and degenerated GP treated tendons (all p < 0.001). Moreover, no difference in fluorescence was detected between the healthy and degenerated control tendons (p = 0.946). GP induced 45% less fluorescence attributed to crosslinks in the degenerated than in the healthy explants (p < 0.001). Furthermore, the distribution of crosslinks induced by GP injection differed between healthy and degenerated tendons. In healthy GP treated tendons, fluorescence attributed to crosslinks was centrally localized and dropped by 32% (p = 0.004) and 69% (p < 0.001) 9mm and 15mm away from the injection site, respectively. In contrast, GP was not confined centrally in degenerated tendons with no significant colorimetric difference at 9mm (-11%, p = 0.278) away from the injection site. For this group, fluorescence dropped as well at 15mm distance by 44% (p < 0.001).

Compared to fluorescence at 590 nm excitation and 645 nm emission, absorption at 590 nm is a less specific indicator for GP-induced crosslinks [34]. Yet, our results for both optical properties were similar and absorption across groups is exhibited in Fig 3B. Healthy GP-injected tendons absorbed significantly more light at 590 nm centrally than degenerated GP treated tendons (p = 0.010), healthy (p < 0.001) and degenerated controls (p < 0.001). For GP treated tendons, the absorption at the injection site was 45% higher in the healthy compared to the degenerated group. Absorption levels did not differ between the healthy and degenerated controls (p = 0.898), and were not significantly different from zero. The relative level of absorption along the tendon was also similar to the distribution of fluorescence. Healthy tendons which were injected with GP exhibited a 37% (p = 0.003) and 69% (p < 0.001) drop 9 mm and 15 mm from the injection site, whereas absorption remained unchanged at 3mm (-10%, p = 0.405). In contrast, absorption was unchanged 3 mm (-8%, p = 0.499) and 9 mm (-9%, p = 0.435) from the injection site in the degenerated group. Absorption dropped by 47% (p < 0.001) in a distance of 15 mm in the degenerated GP group.

### 3.3 Quantification of intratendinous GP concentration

**Fig 4:**
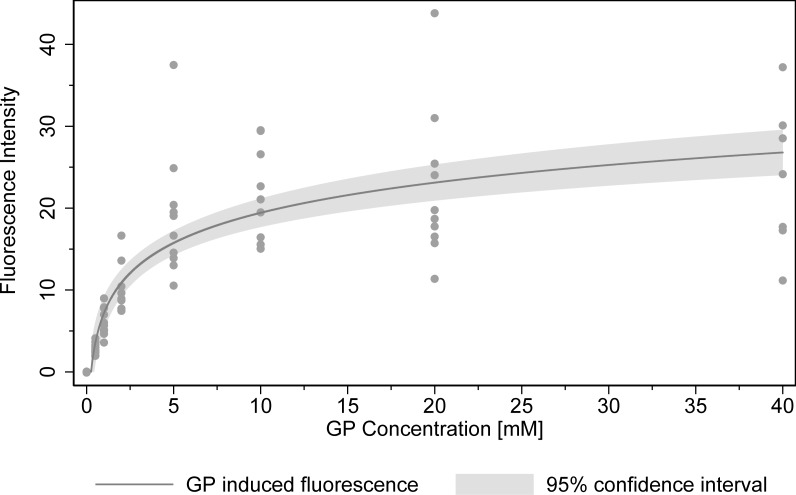
Relationship between GP concentration and fluorescence intensity. GP-induced fluorescence and 95% confidence interval (gray area) were estimated by a linear regression (OLS) using the natural logarithm of GP concentration and a constant as independent variables. Fluorescence intensity is dry weight normalized. Each tendon tissue sample is depicted by a grey dot.

**Fig 5:**
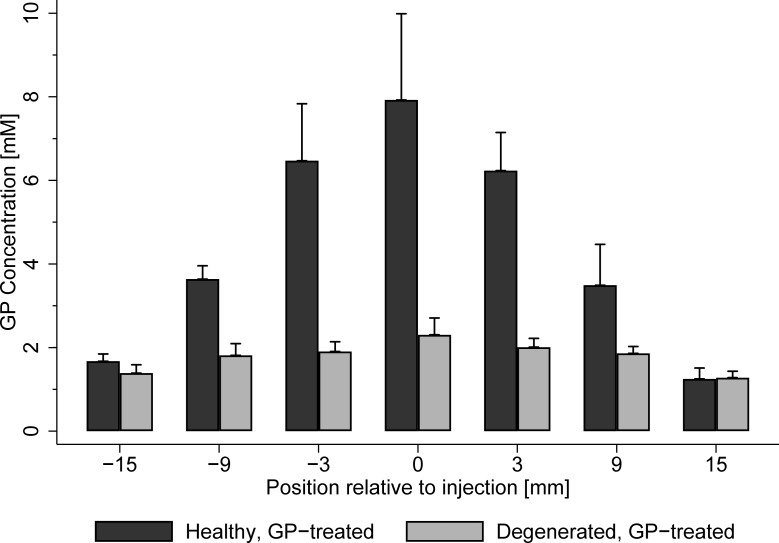
Mean and standard error of estimated tissue GP concentrations for each treatment group. The functional relationship between GP-induced fluorescence (excitation wavelength 590 nm, emission wavelength 645 nm) and GP concentration was established in a separate experiment and by a linear regression (OLS) using the natural logarithm of GP concentration and a constant as independent variables (see **Fig 4**). Observed GP fluorescence in the main experiment served as input to the obtained regression equation, which then yielded the depicted GP tissue concentration.

Fig 4 exhibits the results of the separate experiments conducted to quantify the relationship between GP concentration and induced fluorescence at excitation 590 nm and emission 645 nm. The results suggest a moderate linear relationship between fluorescence and the natural logarithm of GP concentration (R^2adj^ = 0.61). An increase of 1% in GP concentration caused weight adjusted fluorescence to rise by 0.12 (p < 0.001). This relationship was used to estimate the concentration of GP based on the measured fluorescence in the main experiment (Fig 5). For healthy GP treated tendons, GP concentration was estimated to be 7.9 mM at the injection site and drops to 3.6 mM and 1.5 mM 9 mm and 15mm from the center, respectively. Degenerated tendons showed lower GP concentrations of 2.3 mM and 1.8 mM, at the injection site and at a distance of 9 mm, respectively. GP concentration in degenerated tendons 15mm from the center was 1.3 mM and similar to levels in healthy tendons.

## 4 Discussion

This study aimed to investigate the potential of genipin (GP) injection as a treatment of tendinopathy. To our knowledge, this is the first study to investigate the biomechanical effects of GP-treatment by route of injection. The study confirmed the feasibility of minimally invasive injection to strengthen tissue and arrest propagation of pre-rupture damage in tendinopathic lesions. Additionally, this study characterized the resulting degree of intratendinous GP-crosslinking in order to estimate potential cytotoxic effect at a functionally relevant concentration and to characterize dissemination behavior after injection.

Tendinopathy is caused by repetitive microtrauma exceeding the healing capacity of the tendon. Hence, sustained overloading results in damage accumulation and stepwise weakening of the tendon.[11,13,35,36] Continued damage accumulation can even lead to spontaneous rupture of the tendon.[37] Physical rest may prevent further damage at the lesion site, however, might result in deleterious changes to other musculoskeletal structures and is often undesirable by competitive and recreational athletes alike.[13,38]

One approach to break the vicious cycle of damage, inadequate repair and subsequent further damage is to locally strengthen the tendon tissue. Following local tissue stabilization, pain monitored physical activity[22] may provide tenocytes with the appropriate mechanical cues to promote repair at subcritical strain.[39,40]

For this controlled laboratory study, 140 bovine superficial digital flexor tendon explants were either centrally injected with collagenase D to model tendinopathy or with a placebo (PBS) to serve as healthy controls. Injection of collagenase type D disrupts the collagen matrix and is a well-established method to induce degenerative tendon lesions *in-vivo* [7,8,41] and *ex-vivo* [28,29]. The average reduction in ultimate tensile strength of 23% by collagenase injection reported in this study is within the range observed in human post-mortem Achilles tendon explants showing histological degenerative changes.[42] Furthermore, biological and structural changes after collagenase injection in vivo, such as loss of matrix organization, hypercellularity, and increased vascularity, resemble human tendinopathy.[43,44] At 24 hours after induction of chemical degeneration, half of the healthy and degenerated tendons were randomly selected and treated by central intratendinous injection of 0.2 ml of 80 mM GP, while the remaining samples received a placebo (PBS) injection.

In line with our hypothesis, GP injection into chemically induced degenerative tendon lesions significantly increased the ultimate tensile strength by 19% compared to the degenerated control comparable to the strength level of healthy tendons. The GP-treatment however only minimally affected the elastic properties as well as strain behavior of the tendon, implying collagen crosslinking only in proximity to the injection site. The viscoelastic behavior on the other hand was strongly affected by GP as assessed by the relative stress relaxation crosslinking and is in agreement with existing literature. [25,45,46].

The spatially confined effect of GP-injection was confirmed by analyzing the GP-specific optical spectral properties of tendon biopsies taken at different distances from the injection site. Average fluorescence intensity decreased by 69% and 47% within 15mm of the injection site for the healthy and the degenerative tendons, respectively. These findings suggest that GP is able to readily diffuse through degenerated tissue whereas the structural integrity of healthy tissue prevents similar diffusion. Absorption analysis further support these results. This implies that an extensive lesion could be treated by a single high-dose GP injection.

In a separate experiment, GP incubation concentration and corresponding fluorescence was examined. This allowed the quantification of crosslinking induced by GP-treatment and provides an estimate for the respective GP incubation concentration. According to this relationship, the respective incubation concentration was 7.9 mM at the site of injection and decreased to 1.5 mM within 15 mm for healthy tendons. Earlier studies have shown cytotoxicity to be dose dependent on GP concentration within the tissue.[24,46–49] Applying the results from previous in vitro work of Fessel et al.[46] to GP concentration estimates of this study, cell survival is predicted to be 48% and 68% at the injection site and 15mm away, respectively. These cell viability estimates have to be treated with caution as Fessel et al.[46] used isolated tenocytes from equine tendons and the method of GP application differed (incubation vs. injection). Even though our results suggest a spatially limited GP diffusion, further experiments are needed to determine the critical cell survival for repopulation and stabilization of the tissue.

These results are in line with Bellefeuille et al.[24], who examined the local and systemic toxicity of high dose GP injection (355mM) in subcutaneous and intratendinous tissue of horses. There were no signs of inflammatory infiltrates and vasculature appeared normal in all examined tendon tissue samples at short- and long-time follow-up. None of the treated horses showed any apparent discomfort or other adverse clinical effects. Blood cell count and serum chemistry analyses revealed no abnormal findings associated with GP-injection.

Several limitations must be noted. First, while the studied method of GP application showed promising results *ex-vivo*, future work in experimental animal models or in controlled veterinary clinical studies will be necessary to further study the potential of GP-treatment in tendinopathy. The biological effect of GP in general and in an acute or chronic inflammatory response in particular is not fully understood, although limited evidence suggests that GP has no measurable influence on secretion of pro- or anti-inflammatory cytokines.[50] Due to tissue degradation processes, the current model allowed to study only short-term GP-effects. Further, the mechanical testing setup represented a crude approximation of the in vivo mechanical regime of human tendons during daily activities and traumatic failure. Whereas palpation of the tendon samples 24h following collagenase injection indicated collagen damage to be primarily located at the sample mid-portion, the spatial extent of chemical collagen damage could not be assessed objectively. It is therefore unverified that GP-repair covered the entire chemically damaged volume. Knowledge of the local strain field during tensile testing may be informative in this situation. Tendon stretch however is dominated by fibre-to-fibre sliding rendering the interpretation of local strain measurements difficult. [51]Inducing degenerative changes to the tendon tissue using bacterial collagenase rather than using actual human tendinopathic specimens also represents a limitation of this study. More generally, caution is always warranted when extrapolating results obtained from in vitro experiments on animal models to humans.

## 5 Conclusion

This study established proof of concept of a potential clinical method of GP application for the treatment of tendinopathy, provided a comprehensive baseline for GP dosage in the design of future experiments and will be helpful in the interpretation thereof. GP injection into degenerative lesions recovered the mechanical strength of tendons to the level of healthy ones. After injection, GP disseminated within a tendon lesion whereas healthy tissue acts confining. Mechanically functional GP dosage was predicted to yield sufficient cell survival for subsequent repopulation of the affected tissue and warrants further in vivo work.
