# Correction: Assessing the effects of intratendinous genipin injections: Mechanical augmentation and spatial distribution in an *ex vivo* degenerative tendon model

**DOI:** 10.1371/journal.pone.0243256

**Published:** 2020-11-30

**Authors:** The PLOS ONE Staff

The first three authors, Timo Tondelli, Tobias Götschi, and Roland S. Camenzind, should be noted as contributing equally to this work. The publisher apologizes for the error.
